# A functional network of highly pure enteric neurons in a dish

**DOI:** 10.3389/fnins.2022.1062253

**Published:** 2023-01-06

**Authors:** Martial Caillaud, Morgane E. Le Dréan, Adrien De-Guilhem-de-Lataillade, Catherine Le Berre-Scoul, Jérôme Montnach, Steven Nedellec, Gildas Loussouarn, Vincent Paillé, Michel Neunlist, Hélène Boudin

**Affiliations:** ^1^Nantes Université, INSERM, TENS, The Enteric Nervous System in Gut and Brain Diseases, IMAD, Nantes, France; ^2^Nantes Université, CNRS, INSERM, L’institut du Thorax, Nantes, France; ^3^Nantes Université, CHU Nantes, CNRS, INSERM, BioCore, US16, SFR Bonamy, Nantes, France; ^4^Nantes Université, INRAE, IMAD, CRNH-O, UMR 1280, PhAN, Nantes, France

**Keywords:** enteric neuron, enteric synapse, spontaneous activity, lipid-mediated transfection, multi-electrode array, patch-clamp

## Abstract

The enteric nervous system (ENS) is the intrinsic nervous system that innervates the entire digestive tract and regulates major digestive functions. Recent evidence has shown that functions of the ENS critically rely on enteric neuronal connectivity; however, experimental models to decipher the underlying mechanisms are limited. Compared to the central nervous system, for which pure neuronal cultures have been developed for decades and are recognized as a reference in the field of neuroscience, an equivalent model for enteric neurons is lacking. In this study, we developed a novel model of highly pure rat embryonic enteric neurons with dense and functional synaptic networks. The methodology is simple and relatively fast. We characterized enteric neurons using immunohistochemical, morphological, and electrophysiological approaches. In particular, we demonstrated the applicability of this culture model to multi-electrode array technology as a new approach for monitoring enteric neuronal network activity. This *in vitro* model of highly pure enteric neurons represents a valuable new tool for better understanding the mechanisms involved in the establishment and maintenance of enteric neuron synaptic connectivity and functional networks.

## Introduction

The enteric nervous system (ENS) is an intrinsic nervous system located throughout the digestive tract. It is organized in plexi formed by a network of ganglionic structures connected to each other by interganglionic fibers (Schemann and Neunlist, 2004; Kang et al., 2021). These plexi include the myenteric plexus (located between the circular and longitudinal muscle layers) and the submucosal plexus (located between the circular muscle layer and the mucosa) (Brookes, 2001; Schemann and Neunlist, 2004; Kang et al., 2021). The ENS consists of two major cell types; enteric neurons and enteric glial cells (EGCs), derived from enteric neural crest cells that colonize the gut during embryogenesis (Burns and Pachnis, 2009; Kang et al., 2021). A functional neural network has been established following a complex program of developmental maturation extending to the postnatal period (Burns and Pachnis, 2009; Gershon, 2010). Coordinated activity of the enteric neuronal network, through poorly characterized mechanisms regulates major functions of the digestive tract, such as intestinal epithelial permeability, secretion and gut motility (Schemann and Neunlist, 2004). In addition, alterations in the ENS and its functions (known as enteric neuropathies) have been associated with or described as being responsible for gut dysfunction in various gastrointestinal diseases, manifested notably by impaired motility and peristalsis (Burns et al., 2009; Gonzales et al., 2020; Holland et al., 2021).

Neuronal connectivity is a key physiological process involved in ENS function (Nestor-Kalinoski et al., 2022). At the organ level, neuronal connectivity is defined as structural and functional connectivity contributing to neuronal network activity, which is translated at the cellular level by synaptic communication (Park and Friston, 2013). It has been suggested that perinatal development of enteric neuronal connectivity and neurotransmitter expression by neurons play a key role in intestinal motility control (Hao and Young, 2009; Foong et al., 2012). On the other hand, disruption of key processes involved in neuronal connectivity has been reported to impact gastrointestinal motility (Chalazonitis et al., 2011; Sasselli et al., 2013). At the molecular level, key factors known to regulate brain neuronal connectivity, such as members of the Semaphorin and Ephrin families, have been identified to play a putative role in ENS maturation (Gonzales et al., 2020; Bodin et al., 2021). However, a better understanding of the mechanisms regulating neuronal connectivity and its impact on ENS function remains a major problem. This lack of understanding is partly due to the absence of experimental *in vitro* models of enteric neurons. Pure culture models of central neurons have been developed for decades and have proven to be valuable tools for studying fundamental neuronal processes, including dendrite and axon formation, neuronal plasticity, and synaptic activity (Smiałowska and Legutko, 1992; Ray et al., 1993; Yang et al., 2010). Most *in vitro* models of ENS are composed of a mixed culture of enteric neurons, EGCs, and myofibroblasts (Schonkeren et al., 2022), and have provided invaluable data on the identification of trophic factors for enteric neuron development (Heuckeroth et al., 1998; Gougeon et al., 2013) and on the mechanisms regulating neurotransmitter expression (Chevalier et al., 2008). To address glial functions more specifically, pure cultures of EGCs have been developed and used to demonstrate their role in the regulation of intestinal epithelial barrier functions and neuronal development (Van Landeghem et al., 2011; Le Berre-Scoul et al., 2017). However, pure cultures of enteric neurons with dense neuronal networks that are applicable for long-term studies are currently lacking. Several culture models enriched in enteric neurons have been developed, but resulted in cultures that are incompletely pure in enteric neurons (Wu et al., 1999; Sato and Heuckeroth, 2008), with a sparse neuronal network (Bannerman et al., 1988) or containing non-functional neurons (Hawkins et al., 2013).

Commonly used methods for studying enteric neuronal activity are based on calcium (Ca^2+^), voltage-sensitive dye (VSD) imaging, and patch clamp (Vanden Berghe et al., 2001, 2008; Schemann et al., 2002; Bodin et al., 2021). As an emerging electrophysiological technique, the multi-electrode array (MEA) represents a particularly suitable approach for monitoring functional neuronal networks in a prolonged and non-invasive manner, enabling repeated recordings on the same culture over several weeks. Unlike Ca^2+^ and VSD imaging, MEAs does not rely on fluorescent dyes; thus preventing phototoxicity and photobleaching. In addition, MEA is less time-consuming than conventional single-cell electrophysiological techniques such as patch clamp, although it offers a lower degree of spatial resolution. The application of MEA to central nervous system (CNS) neuron cultures has provided a better understanding of neuronal network features, including network dynamics and signal propagation waves (Amin et al., 2016); however, MEA has never been applied to enteric neuron network studies, which might be partly due to the unavailability of enriched neuronal cultures at high density required for this technique. Although a study using a MEA system allowed to characterize the spatial properties of oscillating electrical activity in *ex vivo* mouse ileum, the data collected did not provide a fine analysis of enteric neuronal networks, but rather an overview of a cellular cooperation including muscle, neuronal and interstitial cells (Taniguchi et al., 2013). Finally, a second study recently reported the application of myenteric neuron cultures in MEA chips but without presenting the corresponding recordings (Schulte et al., 2021).

Therefore, this study aimed to develop a long-term culture system for highly pure enteric neurons from rat embryos. We demonstrated that our procedure resulted in highly enriched enteric neuron culture. The main features of enteric neurons were measured by morphological, phenotypic, and functional characterization by immunofluorescence, western blot, electrophysiology, and live imaging approaches. In addition, we found that these cultured enteric neurons established synaptic connections and were organized in a functional neuronal network, as shown by patch-clamp, FM 1-43, and MEA recordings. This novel enteric neuron culture model provides a valuable tool for studies targeting cell-autonomous mechanisms involved in the establishment and maintenance of enteric neuronal connectivity.

## Materials and methods

### Animals

We cultured enteric neurons from rat embryo intestines. We obtained pregnant Sprague-Dawley rats on gestational day 15 (Janvier Labs, Le Genest Saint-Isle, France). The rats were anesthetized with isoflurane and euthanized by cervical dislocation. We collected the embryos and extracted the intestines for cell culture. All animal experiments were performed in accordance with the French Standard Ethical Guidelines for Laboratory Animals.

### Enteric neurons cell culture

Isolation and dissection of the intestines have been previously described (Le Berre-Scoul et al., 2017). Embryonic day 15 (E15) rat intestines were collected and finely dissected in cold Hank’s buffered salt solution (HBSS). Each whole intestine (duodenum to sigmoid colon) was individually placed in a drop of cold HBSS in a Petri dish and cut into 8 pieces of equal length. We placed the 8 pieces of each intestine (referred later as explants) in a 24-well plastic culture plate (Corning^®^-Costar^®^, ref 3524, Merck KGaA, Darmstadt, Germany) previously coated with poly-L-lysine 0.1 mg/mL and covered it with a glia-conditioned culture medium with GDNF 50 ng/ml (R&D Systems – bio-techne, 512-GF-050/CF). We obtained the culture medium from an EGC culture. Enteric glial cell cultures were obtained from ENS primary cultures derived from rat embryonic intestines (Van Landeghem et al., 2011). Briefly, EGC was cultured in DMEM (41965-039- Gibco) supplemented with 10% (vol/vol) heat-inactivated FBS (Eurobio), 10 mM glutamine (25030-024-Gibco), 50,000 IU Penicillin/50,000 IU Streptomycin (15140-122-Gibco). When EGCs were at 50% confluence, DMEM was replaced for 72 h with Neurobasal Medium (ref 21103-049-Gibco) supplemented with B27 (ref: 17504-044-Gibco), 10 mM Glutamine (ref 25030-024-Gibco), 50,000 IU Penicillin/50,000 IU Streptomycin (ref 15140-122-Gibco). This glia-conditioned medium was then filtered through a sterile 0.22 μM PolyEhterSulfone membrane before storage (−20°C) or used. After 5 days of culture (D5), many cells emerged from the explants and covered a large part of the well. We removed the explants using a P1000 pipette, and detached the cells with 300 μl of Accutase per well for 5 min. Accutase was inhibited by adding 4.7 ml of DMEM + FBS and cell suspensions were centrifuged at 1,500 rpm for 5 min at room temperature (RT). The cell pellet was suspended in conditioned-glia medium + GDNF (50 ng/ml) and seeded either in a P24 plate for immunofluorescence, on an 18 mm diameter coverslip (Neuvitro corp., Vancouver, WA, USA) for patch clamp, in a MEA chamber or in Ibidi 8-well plate for Ca^2+^ and VSD imaging, all previously coated with poly-L-lysine 0.1 mg/ml. At D6, an antimitotic (cytosine arabinoside: AraC, 5 μM) was added to the medium to eliminate the remaining glial and muscle cells. Purified neurons were used for functional and morphological tests on D11 and D12.

### Immunostaining

We fixed cells with 4% paraformaldehyde for 15 min. The cells were permeabilized for 5 min in 0.25% Triton-X-100 in PBS at RT. After washing twice in PBS, cells were incubated for 30 min in blocking solution (PBS containing 10% BSA) at RT. Cells were incubated overnight at 4°C with the primary antibodies diluted in PBS containing 3% BSA. The primary antibodies used were as follows: mouse anti-S100β (1:3, Dako, Les Ulis, France), mouse anti-α-smooth muscle actin (α-SMA, 1:2000, Abcam Inc., Cambridge, MA, USA), goat anti-Hu (1:100; Santa Cruz Biotechnology, Santa Cruz, CA, USA), mouse anti-Hu (1:500; Molecular probe), rabbit anti-neuronal nitric oxide synthase (nNOS; 1:500; Enzo), goat anti-choline acetyltransferase (ChAT; 1:200; Millipore), mouse anti-microtubule-associated protein 2 (MAP2; 1:500, Sigma-Aldrich, Saint-Quentin-Fallavier, France), rabbit anti-Synapsin-1 (1:1,000, Cell Signaling), rabbit anti-βIII-tubulin (Tuj1, 1:500, Abcam), and mouse anti-PSD95 (1:200; Thermo Fisher Scientific, Cillebon sur Yvette, France). After washing, we incubated the cells for 90 min at room temperature with the appropriate FITC-conjugated or Alexa 568-conjugated secondary antibody diluted in PBS containing 3% BSA and 0.02% azide. Cells were washed with PBS containing DAPI (Molecular Probes). For each sample, images were acquired using an Axio observer Zeiss microscope (Zeiss, Germany) and Zen software (Zeiss, Germany) and were taken from three randomly chosen fields. Images were quantified using the ImageJ software. Data are presented as mean ± SEM of *n* = 4 culture wells from 4 independent rat embryos.

### Analysis of confocal images

For visualization of synaptic clusters stained by synapsin-1 and PSD95, images were acquired with x60, numerical aperture (NA) 1.4 oil immersion objective using a scanning confocal microscope (NIKON A1 RSi, Nikon, Tokyo, Japan) driven by NIS-Elements software. High resolution 3D confocal images were done accordingly to the Nyquist sampling rate (0.1 μm XY and 0.15 μm Z step) and processed with the Lucy Richardson deconvolution algorithm (NIS-Elements Software). In each sample, the two fluorophores (FITC or Cy5) were recorded separately using sequential scanning to eliminate the possibility of overlapping emission.

### ENS rat primary culture

Primary cultures of rat ENS (mixed culture with muscle, glia, and neuronal cells) were generated and cultured as previously described (Chevalier et al., 2008). Briefly, the intestines of rat embryos at E15 (Sprague–Dawley rat, Janvier Laboratories SA, Le Genest-St-Isle, France) were finely diced and digested at 37°C for 15 min in DMEM-F12 (Gibco, Life Technologies, Cat# 31330-038) with 0.1% (w/v) trypsin (Sigma, Cat# T1426). The trypsin reaction was stopped by a medium containing 10% (v/v) fetal bovine serum (Sigma, Cat# F2442) and then treated with 0.01% (w/v) DNAse I (Sigma, Cat# DN25) for 10 min at 37°C. After trituration, we centrifuged the cells at 750 rpm for 10 min, and seeded them in 48-well plates previously coated for 30 min at 37°C with a solution of 0.5% (w/v) gelatin (Sigma, Cat# G6144). After 24 h, the medium was replaced with a serum-free DMEM-F12 medium containing 1% (v/v) of N-2 supplement (Life Technologies, Cat# 17502-048).

### Western blot

Enteric nervous system and highly pure enteric neuron cultures were lysed in RIPA buffer (20-188, Millipore), completed by phosphatase (phosphatase inhibitor cocktail 3, P0044, Sigma-Aldrich) and protease inhibitors (protease inhibitor cocktail, 11873580001, Roche), and then sonicated. Total protein content was quantified using the Pierce BCA protein assay kit (23225, Thermo Fisher Scientific) before adding an adjusted volume of sample reducing agent (NP0009, Invitrogen, Thermo Fisher Scientific) and LDS Sample Buffer (NP0008, Thermo Fisher Scientific).

Equal amounts of lysate were separated using NuPage 10% Bis-Tris Gels (Thermo Fisher Scientific), together with the 2-(N-morpholino) ethanesulfonic acid/sodium dodecyl sulfate (MES-SDS, B0002, Thermo Fisher Scientific) running buffer, before electrophoretic transfer to nitrocellulose membranes using the iBlot™ Dry Blotting System (Thermo Fisher Scientific). Membranes were then blocked for 1 h at room temperature in TBS with 0.1% (v/v) Tween-20 and 5% (w/v) non-fat dry milk and incubated overnight at 4°C with the following primary antibodies: mouse monoclonal anti-Tuj1 (1:1,000; T8660, Sigma), mouse monoclonal anti-PGP 9.5 (1:1,000, MA1-83428, Thermo Fisher Scientific), mouse monoclonal anti-GFAP (1:1000, GA5, IF03L, Calbiochem), rabbit polyclonal anti-α-SMA (1:1000, ab5694, Abcam). To confirm equal protein loading, the membranes were probed with a mouse monoclonal anti-β-actin antibody (1:10,000; A5441, Sigma). Bound antibodies were detected with horseradish peroxidase-conjugated anti-rabbit or anti-mouse antibodies (1:5,000; Life Technologies, Thermo Fisher Scientific) and visualized by enhanced chemiluminescent detection using Clarity ECL substrate (1705061, Biorad). The immunoreactive bands were imaged using laser-scanning densitometry and Image Lab software (Bio-Rad).

### Transfection

We collected explant-derived cells after Accutase treatment on D0, resuspended in 2 ml of conditioned glia medium, and transfected them by incubation for 1h at 37°C with 1 μl Lipofectamine^®^ (Invitrogen) and 1 μg of cDNA encoding membrane-targeted GFP (mGFP) cDNA [GAP-GFP(S65T), a gift from Dr. K. Moriyoshi, Kyoto University, Japan (Moriyoshi et al., 1996). The cell suspension was then distributed into four wells of a 48-well plastic culture plate. On D3, cultures were fixed with 4% paraformaldehyde for 15 min for microscopic analysis.

### Morphological analysis of transfected neurons

Transfected neurons were analyzed by microscopy (Axio Observer Zeiss, Germany; Objectif X20). The transfection rate was calculated by counting the number of mGFP + neurons in a 48-well culture plate, normalized by the number of Hu + cells in the same well. We counted cells using the multi-point tool in the ImageJ software. The morphological classification of transfected neurons was based on the number and length of axons arising from the cell body as well as the diameter of the cell body. In addition, the dendritic morphology was scored as lamellar or filamentous according to previous studies (Foong et al., 2012). A total of 183 GFP-positive neurons have been analyzed from *n* = 6 culture wells from three independent rat embryos.

### Synaptic vesicle recycling

Synaptic vesicle endo/exocytosis recycling by enteric neurons was studied using FM1-43 imaging (Molecular Probes) in Ibidi 8-well plate (μ-Slide 8 Well, #80826, Gräfelfing, Germany). Neurons were loaded for 60 s at 37°C with 5 μg/ml of FM 1-43 in 1 ml of stimulation buffer composed of 31.5 mM NaCl, 90 mM KCl, 2 mM MgCl_2_, 2 mM CaCl_2_, 25 mM HEPES, 30 mM glucose solubilized in H_2_O at pH 7.4. Cells were washed for 15 min at 37°C in Ca^2+^-free washing buffer (119 mM NaCl, 2.5 mM KCl, 4 mM MgCl_2_, 25 mM HEPES, and 30 mM glucose solubilized in H_2_O at pH 7.4) to avoid unloading by spontaneous activity. Finally, Ca^2+^-free washing buffer was removed and replaced by stimulation buffer. The cells were imaged for 60 s at 37°C (in the stimulation buffer). Dynamic experiments (30 fps) were performed using a resonant scanning confocal microscope (NIKON A1 RSi, Nikon, Tokyo, Japan) with an oil-immersion objective (×63, NA, 1.40). Excitation and emission wavelengths were respectively set at 488 and 595 nm, accordingly to FM1-43 fluorescence spectrum. We analyzed images using the ImageJ software. The percentage of fluorescence after 60 s was calculated for each cluster using the formula: ((F_0s_-F_60s_)/F_0s_)/×100 where F_0s_ is the fluorescence intensity at 0 s and F_60s_ at 60 s. Previous studies conducted in hippocampal neurons have described different populations of destaining events that might reflect different mode of exocytosis (Richards et al., 2005). We thus classified the FM1-43 synaptic clusters based on their percentage of remaining fluorescence at 1 min as compared to fluorescence intensity measured at t0. We have arbitrary defined 3 types of synaptic clusters according to the remaining FM1-43 fluorescence at 1 min. The first type was defined by synaptic clusters retaining a high level of fluorescence intensity (≥80% of cluster’s intensity at t0s), the second type by synaptic clusters retaining a low level of fluorescence intensity (≤50%) and the last type by synaptic clusters retaining an intermediate level of fluorescence intensity (comprised between 80 and 50%). A total of 154 FM1-43-positive clusters have been analyzed in three culture wells from three independent rat embryos. To assess the impact of putative photobleaching on fluorescence, cultures loaded with FM1-43 were recorded for one minute in washing buffer. In these non-stimulated conditions, we observed that the remaining FM1-43 fluorescence intensity was in average 99.1 ± 0.6% (SEM) at 1 min as compared to fluorescence intensity measured at t0. Analysis performed on two culture wells from two independent rat embryos.

### Calcium imaging

We performed Ca^2+^ imaging using Cal-520, AM dye (Abcam, Cambridge, UK) in Ibidi 8-well plates (μ-Slide 8 Well, #80826, Gräfelfing, Germany). Neurons were loaded into glia-conditioned medium supplemented with Cal-520 AM dye (5 μM) for 60 min at 37°C in a cell culture incubator. High-frame-rate (60 fps) images were recorded using a resonant scanning NIKON A1 RSi confocal microscope (Nikon Instruments, Champigny sur Marne, France) with a dedicated oil immersion objective (×63, NA, 1.40) and a temperature (37°C)/CO_2_ (5%) controller. The dye was excited by a laser source at 488 nm and the fluorescence signal was recorded at 520 nm. Video acquisition was performed using the Nikon imaging software NIS-Elements. We analyzed the images using the ImageJ software. The fluorescence intensity was measured in region of interest (ROI) delimitating neuronal cell bodies for spontaneous activity and upon pharmacological stimulations. Data were represented as changes in fluorescence intensity according to the formula ΔF = F/F_0_, where F_0_ is the minimum fluorescence intensity. The different Ca^2+^ transient patterns were defined according to the criteria previously reported (Margiotta et al., 2021) i.e., isolated (simple peaks), tightly clustered (compound peaks) and moderately clustered Ca^2+^ transients (a mixture of simple and compound peaks). The analyses were carried out on a total of 70 neurons from n = 4 cultures from 4 independent rat embryos. Cells were stimulated or inhibited with the following pharmacological compounds veratridine (30 μM, Sigma), Di-MethylPhenylPiperazinium (DMPP, 10μM, Sigma), 2 (3)-O-(4-benzoylbenzoyl)-Adenosine TriPhosphate (BzATP, 100 μM, Sigma), Potassium Chloride (KCl, 75 mM, Sigma), and Tetrodotoxine (TTX, 0.5 μM, Sigma). The analyses were carried out on a total of 44 neurons from *n* = 4 culture wells from 4 independent rat embryos.

### Voltage-sensitive dye imaging

We perfomed voltage-Sensitive Dye (VSD) imaging using the FluoVolt™ membrane potential kit (Thermo Fisher Scientific, Waltham, Massachusetts, USA) in Ibidi 8-well plates (μ-Slide 8 Well, #80826, Gräfelfing, Germany). Neurons were loaded into glia-conditioned medium supplemented with the 1000X FluoVolt™ dye (1/1000) and the 100X Power Load Concentrate (1/100) for 15–20 min at 37°C in an incubator. Similarly, for calcium imaging, the dye was excited at 488 nm, and the fluorescent signal recorded at 520 nm using a resonant scanning confocal microscope (NIKON A1 RSi) at 37°C and 5% CO_2_. We acquired images at 500 fps and analyzed them using the ImageJ software. Fluorescence intensity data were measured in an ROI of inter-cluster fibers. The fibers were classified according to their size with the following criteria: small-caliber fibers = width ≤1 μm and large-caliber fibers = width ≥ 1μm. Spontaneous activity data were presented as changes in fluorescence intensity according to the formula ΔF = F/F_0_, where F_0_ is the minimum fluorescence intensity. The analyses were carried out on a total of 15 fibers from *n* = 4 culture wells from four independent rat embryos.

### Patch clamp electrophysiology

We transferred isolated enteric neurons to a recording chamber and perfused them continuously with an external solution containing (in mM):135 NaCl, 5.4 KCl, 0.33 NaH_2_PO_4_, 1 MgCl_2_, 2 CaCl_2_, 5 HEPES, and 5 Glucose (pH adjusted to 7.4 with NaOH, and osmolarity adjusted to 300 mosmol) at room temperature. Neurons were visualized using an Olympus microscope (BX51WIF; Olympus Corporation, Japan) and an X40 water-immersion objective (Olympus U-CAMD3; Olympus Corporation, Japan). Recordings were performed using borosilicate pipettes (glass capillaries GC150TF-10, Harvard apparatus, USA) prepared with a micropipette puller (PC-10, Narishige Co, LTD, Japan). Electrodes (7-8 Mohm) were filled with a solution containing the following (100 mM K-aspartic acid, 30 mM KCl, 1 mM MgCl2, 4.5 mM ATP, 10 mM HEPES, and 6 mM EGTA (pH adjusted to 7.2 with KOH, and osmolarity was adjusted to 300 mosmol).

We recorded data using a HEKA amplifier (EPC10 USB double) and the PATCHMASTER software (HEKA Instrument Inc., New York, USA). The signals were digitized at 1 kHz. Whole-cell recordings were corrected for a junction potential of +18 mV. The series resistance was monitored throughout the experiment using a brief voltage step of −10 mV at the end of each recording. In total, 23 neurons were patched (from *n* = 5 cultures from 5 independent rat embryos) but only 15 neurons had an analyzable action potential (AP).

### Data analysis

#### Cell-attached

We performed cell-attached patch-clamp recordings to record the spontaneous firing activity of isolated enteric neurons while preserving the cytoplasmic contents. Cell-attached recordings were performed exclusively in the voltage-clamp mode, taking care to tune the injected current to 0 pA for 5 min (Perkins, 2006). The firing frequencies (Fi) were averaged from 10 continuous recordings (at least 2 min of continuous recordings) from each neuron.

#### Whole-cell patch clamp

Current-clamp: we applied progressive current injections, from a basal current of −150 pA, and increased by 500 ms steps of 10 pA at 0.1 Hz, until AP firing. We analyzed action potential parameter recordings following a current injection of +30 pA above rheobasis. We measured the I/V relationship and active membrane properties using Fitmaster (HEKA Elektronik, Germany): membrane potential, amplitude of action potential, rise and decay time duration, amplitude of hyperpolarization, duration to reach hyperpolarization amplitude, and frequency of action potential.

Voltage-clamp: Neurons were then voltage clamped at −60 mV, and the minature post-synaptic currents (mPSCs) were recorded and analyzed during 250 s recording segments. We identified the (mPSCs) using a semi-automated amplitude threshold-based detection software (Mini Analysis 6.0.7 Program, Synaptosoft, Fort Lee, NJ, USA).

#### Multi-electrode array electrophysiology

We performed MEA recordings using the MEA2100-Mini-System (MultiChannel Systems), which includes the signal collector unit USB-ME64-system equipped with a FA60SBC filter amplifier (total gain = 1,100), a power supply (PS40W), and a temperature controller (TC02). Standard 60-electrode MEA chips (60MEA200/30iR-Ti) were pre-coated with 0.1 mg/ml of poly-L-lysine and 0.1 mg/ml poly-L-ornithine overnight at room temperature. After rinsing the chips, enteric neurons were plated onto the MEA chips and maintained for 28 days in an incubator at 37°C and 5% CO_2_ before recording. During recordings (10 min per MEA chip), plates were kept on a heated stage maintained at 37°C and covered with a plate lid to prevent evaporation of the medium. Spontaneous network activity from rat enteric neuron cultures on MEA chips was acquired using the MultiChannel Experimenter software (version 2.17.8.21078). We analyzed the data using a MultiChannel Analyzer (version 2.18.0.21200). The recordings were digitized at 50 kHz. During offline analysis, a digital 2nd order high pass Bessel filter with a cutoff of 100 Hz was applied. The peak detection threshold was set with an average noise of five times the signal standard deviation on a per-channel basis (dead time of 3 ms, pre-trigger of 1 ms, and post-trigger of 2 ms). Bursts were analyzed using the following parameters: maximum interval of 50 ms to start/end the burst, minimum interval of 100 ms between bursts, minimum burst duration of 50 ms, and at least four spikes in a burst. Data are presented as mean ± SEM of *n* = 3 chips from 3 independent rat embryos.

### Statistical analysis

All data are expressed as mean ± SEM (Standard Error of the Mean). Normality and equal variance were verified using the Shapiro–Wilk and Brown–Forsythe tests, respectively. Data were then compared using a t-test or one-way analysis of variance (ANOVAs), followed by *post-hoc* Tukey’s comparison test. All statistical analyses were performed using the GraphPad Prism statistical software (GraphPad Software, Inc., La Jolla, CA, USA). Statistical significance was set at *p* < 0.05.

## Results

### Establishment of a highly pure neuron culture

**FIGURE 1:**
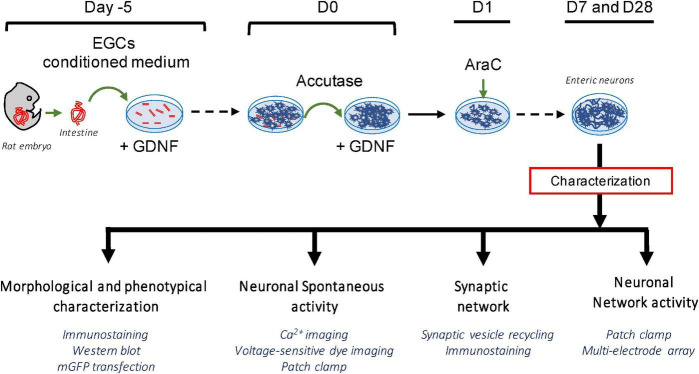
Schematic summary of the experimental protocol for the establishment of a highly pure culture of enteric neurons.

The establishment of a highly pure culture of enteric neurons was based on a novel two-step protocol applied to rat E15 embryos as summarized in Figure 1. First, the gut explants were cultured for 5 days (D-5 to D0), and the cells that migrated from the gut explant recovered by mild accutase treatment on D0 and plated in a culture dish for seven additional days to obtain a dense neuronal network at D7 (Figure 1).

**FIGURE 2:**
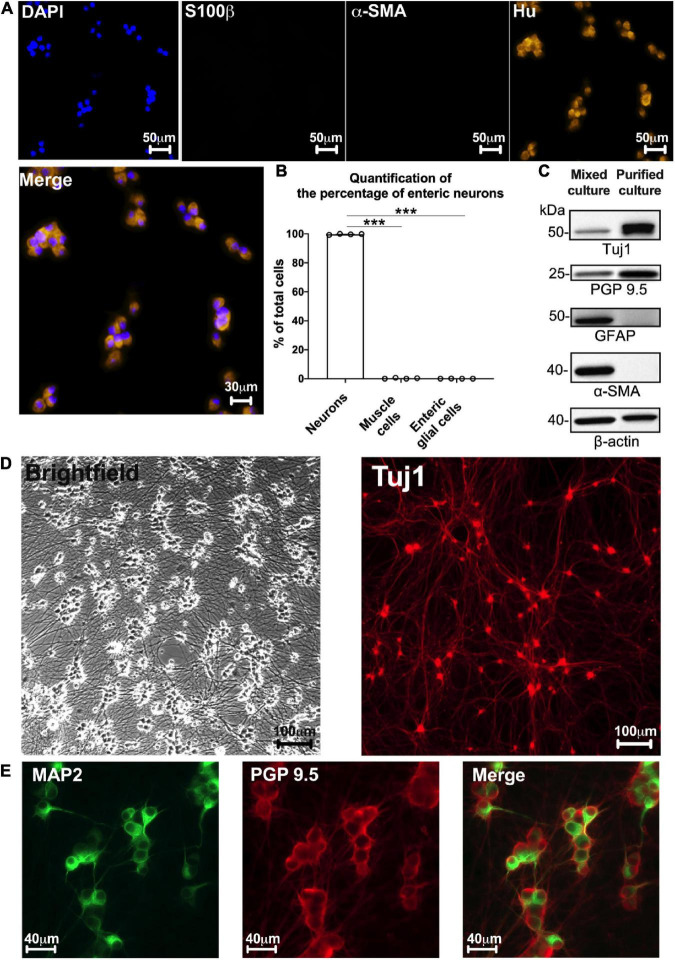
Highly pure culture of rat enteric neurons. **(A)** Staining for cell nucleus (DAPI) and immunostaining for enteric neurons (Hu), glial cells (S100b), and myofibroblasts (α-SMA) at D7 culture. **(B)** Quantification of the percentage of enteric neurons, glial cells and myofibroblasts in cultures at D7 (Data are presented as mean ± SEM from 644 neurons analyzed from *n* = 4 culture wells from 4 independent rat embryos). **(C)** Representative western blot from ENS mixed cell culture (muscle, glia, and neuronal cells) and highly pure culture of enteric neurons (*n* = 4 culture wells from 4 independent rat embryos). **(D)** Brightfield and immunostaining for TUJ1 in culture at D7. **(E)** Double immunostaining of enteric neurons at D7 with the somatodendritic marker MAP2 (green) and PGP 9.5 (red) (*n* = 3 culture wells from 3 independent rat embryos). Results were compared using one-way ANOVA and *post-hoc* Tukey’s test (***p < 0.001).

Besides neurons, other putative cell types derived from gut explants could be EGCs and muscle cells. To determine the proportion of each cell type, we performed triple immunolabeling for Hu, S100β, and α-SMA to specifically label neurons, EGCs, and muscle cells, respectively (Figure 2A). At D7, 99.7 ± 0.14% of total cells (identified using DAPI staining) were neurons [Hu-immunoreactive (IR)], 0.25 ± 0.15% were muscle cells (α-SMA-IR) and only 0.05 ± 0.02% were EGCs (S100β-IR) (neurons vs. muscle cells: *p* < 0.001; neurons vs. EGCs: *p* < 0.001, *n* = 4 culture wells from four independent rat embryos, Kruskal–Wallis) (Figure 2B). Neurons are mainly organized as clusters composed of 5–8 neuronal cell bodies. To further confirm the purity of neurons in our culture model, we performed Western blot (WB) using antibodies against the neuronal proteins Tuj1 and PGP 9.5, the glial protein GFAP, and the muscle cell protein α-SMA. As a control, ENS mixed cultures were also analyzed by WB with the same markers (Figure 2C). We only detected Tuj1 and PGP 9.5 expression in our neuron culture model, while the markers of all the cell types were observed in the ENS mixed culture (Figure 2C). Regarding the network organization, neuronal cell bodies were present at high density and showed a profuse network of nerve processes (Figure 2D). These were later characterized as axons when labeled with Tuj1 and PGP 9.5, respectively (Figures 2D, E), and as dendrites when labeled with MAP2 (Figure 2E). These results show that the experimental design developed in this study resulted in a primary culture model composed of neurons.

### Morphological and phenotypical characterization of enteric neurons

**FIGURE 3:**
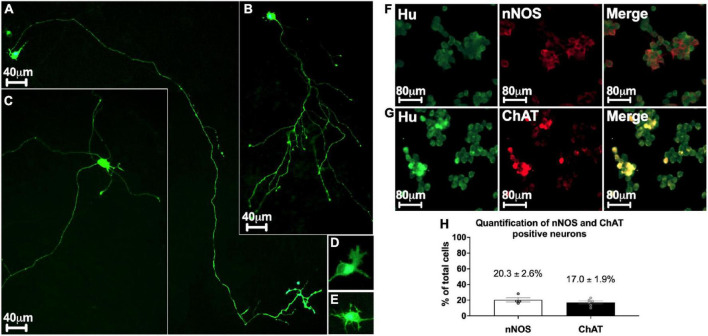
Morphological and phenotypical characterization of enteric neurons. **(A–E)** mGFP transfected enteric neurons at D3. **(A,B)** Monoaxonal enteric neurons with a small-diameter cell body. Monoaxonal neurons can be sorted into two sub-types based on axon morphology: long axonal process with sparse and short ramifications **(A)** or medium-length axon with numerous ramifications **(B)**. **(C)** Multiaxonal neurons exhibiting more than 2 axons of medium to short length and a large-diameter cell body (183 neurons analyzed from n = 4 culture wells from 4 independent rat embryos). **(D,E)** Neuron with lamellar **(D)** and filamentous **(E)** dendrites (110 neurons analyzed from *n* = 3 culture wells from 3 independent rat embryos). **(F,G)** Double immunostaining of enteric neurons with the neuronal cell body marker Hu (green) with either nNOS (red, F) or ChAT (red, G) at D7. **(H)** Percentage of neurons immunostained with neuronal nNOS and ChAT at day 7. Data are presented as mean ± SEM from 886 neurons analyzed from *n* = 4 culture wells from 4 independent rat embryos.

Several subtypes of enteric neurons have been identified, based on their morphology and neurochemical phenotypes. The morphological criteria concern the size of the cell body, presence of dendrites, and axonal architecture. To assess the morphology of enteric neurons in our culture model, we transfected cells with an mGFP plasmid on D0 and analyzed their morphology on D3. We found a mean of 183.60 ± 18.25 neurons expressing mGFP in a 48-well culture plate, corresponding to a transfection rate of 0.26 ± 0.03%. This small fraction of transfected neurons enabled the visualization of the entire morphology of a single neuron in a dense neuronal network (Figure 3). Three distinct morphological classes of transfected neurons were observed. The first class describes monoaxonal neurons with a small cell body diameter (∼20 μm) (Figures 3A, B). This first class, predominant, represent 94.7 ± 0.9% of transfected neurons and can be divided in 2 sub-types. Thus, we distinguish neurons with a long axonal process with sparse and short ramifications (Figure 3A) or a medium-length axon with numerous ramifications along the axonal process (Figure 3B). The second class (2.5 ± 0.5% of transfected neurons) is composed of multiaxonal neurons characterized by >2 axons of medium to short length and a large-diameter cell body (40–45 μm) (Figure 3C). Finally, a third and intermediate class was observed, with neurons exhibiting two axons and a small-diameter cell body (∼20 μm) (2.8 ± 0.5% of transfected neurons) (*n* = 6 culture wells from 3 independent rat embryos). Interestingly, the first two morphological classes described above are reminiscent of types I and II of Dogiel’s classification of enteric neurons, respectively. In addition, the morphological analysis of the dendrites showed that at D3, 23.2 ± 4.5% of them had a lamellar morphology (Figure 3D), 52.1 ± 5.5% a filamentous morphology (Figure 3E), and 24.7 ± 1.7% a combination of both morphologies (*n* = 3 culture wells from 3 independent rat embryos).

Based on neurochemical criteria, the most represented neurons in the ENS are nitrergic and cholinergic neurons. To assess the neurochemical phenotype of enteric neurons in highly pure culture, cells were immunolabelled for nNOS and ChAT to identify nitrergic (Figure 3F) and cholinergic (Figure 3G) neurons, respectively. We found that 20.3 ± 2.6% of total neurons, as determined by Hu staining, were nNOS-positive neurons and 17 ± 1.9% were ChAT-positive neurons (Figure 3H).

Taken together, these results indicate that our culture model is composed of cells with morphological and phenotypic characteristics of enteric neurons.

### Functional characterization of enteric neurons

**FIGURE 4:**
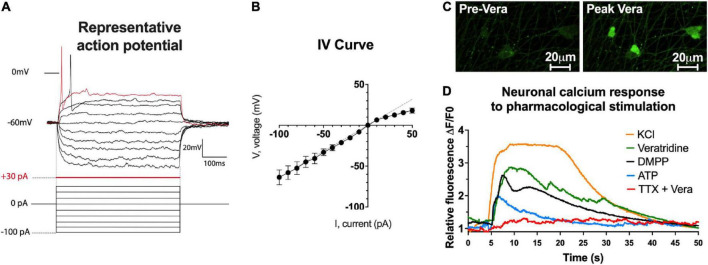
Functional characterization of enteric neurons. **(A)** Raw traces show individual voltage responses to series of 500 ms current applied at 0.1Hz, with steps of 10 pA until neuron firing. **(B)** Average steady-state I/V relationship of recorded enteric neurons (15 neurons analyzed from *n* = 5 cultures from 5 independent rat embryos). **(C,D)** Fluorescence images **(C)** and representative plot of Ca^2+^ response **(D)** evoked by 75 mM KCl, 10 μM DMPP, 100 μM BzATP, 30 μM veratridine and 30 μM veratridine + 0.5 μM TTX (44 neurons analyzed from *n* = 4 cultures from 4 independent rat embryos).

One of the fundamental features of neurons is to be electrically excitable cells with the ability to produce APs. To determine the electrophysiological characteristics of APs generated by highly pure enteric neuron cultures, we performed whole-cell patch clamp recordings (Figures 4A, B). The data obtained on the active and passive membrane properties of neurons are detailed in Table 1 and Figures 4A, B. The electrophysiological data showed that the APs of enteric neurons have on average a rheobase of 37.7 ± 5.9 pA, a threshold of −25.1 ± 1.6 mV, an amplitude of 45.7 ± 3.0 mV, and a duration of 6.9 ± 0.7 ms. In addition, a break in the linearity of the I/V curve, represented by the linear regression line, was observed after depolarization (Figure 4B). This result suggests outward rectification corresponding to the filtration of the input information.

**TABLE 1:** Summary of passive and active membrane properties of enteric neurons recorded in whole-cell patch clamp configuration.

Action potential (AP) properties			
	**MEAN**	**SEM**	***N***
Ri steady state (Go)	662.85	76.65	15
Rhéobase (pA)	37.66	5.91	15
AP threshold (mV)	−25.13	1.57	15
AP delay (ms)	78.90	20.16	15
AP amplitude (mV)	45.68	2.98	15
AP rise (ms)	3.16	0.38	15
AP decay (ms)	3.74	0.31	15
AP duration (ms)	6.91	0.66	15
fAHP duration (ms)	10.20	1.34	15
fAHP amplitude (mV)	−20.36	1.33	15
sAHP duration (ms)	110.23	21.41	15

Next, the ability of enteric neurons to respond to ion channel activators and neurotransmitter receptor agonists was investigated at D7 using Ca^2+^ imaging based on the Ca^2+^ probe Cal520-AM (Figures 4C, D). The addition of veratridine (30 μM), an activator of voltage-dependent Na^+^ channels, induced a progressive and high increase in intracellular Ca^2+^, which remained at a high level for 35 s of recording with a percentage of responding cells of 87.5 ± 7.9% (Figures 4C, D). Pre-treatment with the voltage-dependent Na^+^ channel blocker TTX (0.5 μM) prevented veratridine-induced response (Figure 4D). Depolarization induced by KCl (75 mM) resulted in a rapid and elevated intracellular Ca^2+^ response on 97.2 ± 2.8% of neurons (Figure 4D). Regarding the activation of neurotransmitter receptors, the nicotinic cholinergic receptor agonist DMPP (10 μM) and the purinergic receptor agonist BzATP (100 μM) induced a rapid and transient increase in the intracellular Ca^2+^ response with 63.5 ± 10.4% and 48.5 ± 12.5% neurons responding to pharmacological stimulation, respectively (Figure 4D). These data suggest that enteric neurons in culture are sensitive to activation/inhibition of voltage-dependent Na^+^ channels and depolarization, and that they express functional cholinergic and purinergic receptors.

### Activity of enteric neurons within the network

**FIGURE 5:**
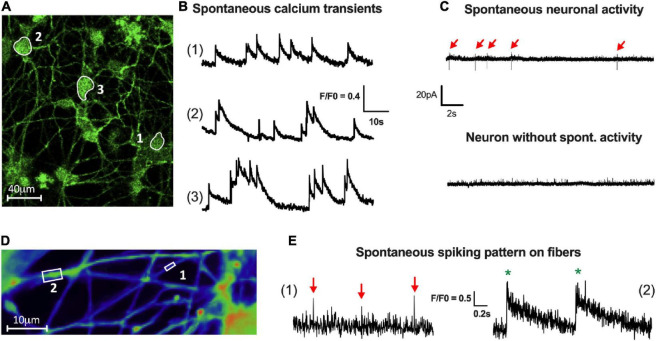
Activity of enteric neurons within the network. **(A,B)** Fluorescence images **(A)** of a field containing neuronal cell bodies with spontaneous activity (1, 2 and 3). **(B)** Representative traces of three types of spontaneous Ca^2+^ activity defined as isolated (1), moderately clustered (2) and tightly clustered transients (3) (70 neurons analyzed from *n* = 4 cultures from 4 independent rat embryos). **(C)** Representative traces of spontaneous spiking pattern in patch clamp cell-attached configuration (15 neurons analyzed from *n* = 5 cultures from 5 independent rat embryos). **(D)** Fluorescence images of a field containing neuronal fibers of small (boxed region 1) and large (boxed region 2) caliber with spontaneous activity visualized by VSD imaging. **(E)** Representative traces of isolated spikes (red arrow) on small caliber fibers (1) or compound potentials (green*), composed of several spikes, on large caliber fibers (2) (15 fibers analyzed from *n* = 4 cultures from 4 independent rat embryos).

The establishment of a functional neuronal network is characterized by the spontaneous activity of the neurons. This neuronal activity can be measured by Ca^2+^ imaging through the analysis of spontaneous Ca^2+^ transients, as previously described (Boesmans et al., 2008; Margiotta et al., 2021). Here, we assessed the ability of enteric neurons at D7 to generate spontaneous intracellular calcium transients using Ca^2+^ imaging. We found that cultured enteric neurons exhibited spontaneous activity in the cell body, with an average calcium transient frequency of 0.88 ± 0.15 Hz (Figures 5A, B). In addition, several Ca^2+^ transient patterns were observed, with isolated (1), moderately clustered (2), and tightly clustered transients (3) (Figure 5B).

Spontaneous electrical activity was also studied using cell-attached patch clamp on neuronal cell bodies (Figure 5C). The results showed that 65% of the neurons exhibited a spontaneous activity with an average AP frequency of 0.15 ± 0.04 Hz (Figure 5C).

Finally, to assess spontaneous activity at the level of the neuronal fiber network, we used a voltage-sensitive probe approach with FluoVolt™ dye. Using VSD imaging, enteric neurons showed the ability to generate spontaneous spikes, which are present along the fibers, with an average frequency of 0.19 ± 0.05 Hz (Figures 5D, E). Moreover, depending on the size of the fibers analyzed, we were able to obtain either isolated spikes on small-caliber fibers (1) or compound potentials (composed of several spikes) on large-caliber fibers (2) (Figures 5D, E).

The observation of spontaneous activity of enteric neurons measured on cell bodies and nerve fibers suggests the existence of connectivity between enteric neurons operating through neuronal cell bodies and fiber network.

### Synaptic connectivity of enteric neurons

**FIGURE 6:**
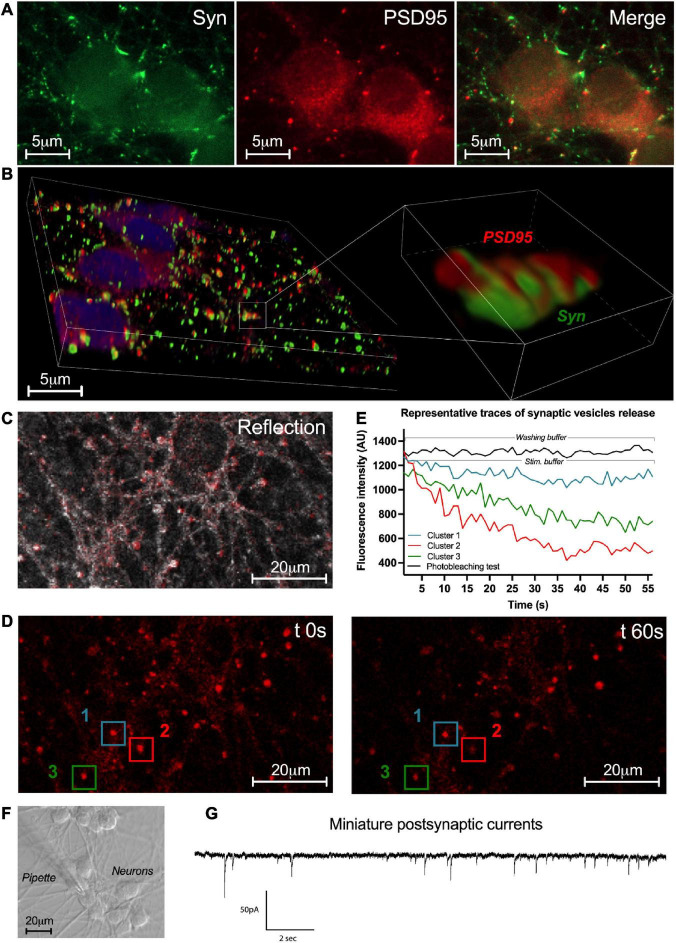
Synaptic network activity of enteric neurons. **(A)** Double immunostaining of enteric neurons at D7 with the pre-synaptic marker synapsin I (Syn; green) and the post-synaptic marker PSD95 (red) (*n* = 3 culture wells from 3 independent rat embryos). **(B)** 3D reconstructed confocal microscopy images of cultured neurons immunolabeleled for synapsin 1 (green) and PSD95 (red) with a higher magnification of the boxed region. **(C)** Representative image of the enteric neuron culture in reflection microscopy. **(D)** Fluorescence images of a field containing clusters measured at t 0s and t 60s after KCl stimulation. **(E)** Plot of FM1-43 fluorescence intensity analyzed from a representative cluster recorded in washing buffer and 3 representative clusters recorded in stimulation buffer of the enteric neuron culture at D7 (154 clusters analyzed from *n* = 3 culture wells from 3 independent rat embryos). **(F)** Representative image of enteric neurons in light microscopy and the patch clamp pipette used for the whole-cell configuration. **(G)** Representative trace of miniature post-synaptic currents (mPSCs) recorded from enteric neurons at D7 (10 neurons analyzed from *n* = 5 culture wells from 5 independent rat embryos).

To further study the properties of enteric neuron connectivity within the network, the presence of synapses was first assessed at D7 by immunofluorescence with the pre- and post-synaptic markers synapsin-1 and PSD95, respectively (Figures 6A, B). Synapsin-1 was distributed in clusters around the neuronal perikarya and along the axonal extensions (Figure 6A). The post-synaptic protein PSD95 was detected as clusters in neuronal cell bodies and throughout the fibers in proximity to neuronal cell bodies (Figure 6A). To further analyze the spatial relationships between synapsin1 and PSD95, we performed confocal microscopy imaging with 3D reconstruction and quantified the number of apposed synapsin1 and PSD95 clusters (Figure 6B). We found that approximately 33 ± 10% of total synapsin1 clusters were apposed to PSD95 clusters, suggesting that a fraction of synapses formed between enteric neurons contain synapsin 1 and PSD95 as pre- and post-synaptic proteins, respectively.

To assess the functionality of the synapses previously revealed with synaptic markers synapsin-1 and PSD95, labeling of synaptic vesicle endo/exocytosis recycling was performed by FM1-43 imaging (Figures 6C–E). Enteric neurons were exposed to FM1-43 dye while stimulated by depolarization with the stimulation buffer (KCl 75 mM) to allow FM1-43 loading by endocytosis. After washout of excess dye with washing buffer, active synaptic clusters appeared as bright red fluorescent spots throughout the neuronal network (Figure 6C). After stimulation with the stimulation buffer, synaptic vesicle exocytosis was assessed by measuring the remaining FM1-43 fluorescence intensity at 1min (Figures 6D, E). We observed three types of synaptic clusters according to the fration of FM1-43 remaining fluorescence as measured at 1 min. The first type was defined by synaptic clusters retaining a high level of fluorescence intensity (≥ 80% of cluster’s intensity at t0s) corresponding to clusters with low level of synaptic vesicle release activity (Figures 6D, E cluster 1). The second type was defined by synaptic clusters retaining a low level of fluorescence intensity (≤50%) corresponding to clusters with high level of synaptic vesicle release activity (Figures 6D, E cluster 2). The last type was defined by synaptic clusters retaining an intermediate level of fluorescence intensity (comprised between 80% and 50%) corresponding to clusters with intermediate level of synaptic vesicle release activity (Figures 6D, E cluster 3). These three types of synaptic clusters represent respectively, 42.7 ± 19.6%, 15.1 ± 15.1%, and 42.2 ± 4.9% of analyzed synaptic clusters (154 clusters were analyzed from *n* = 3 culture wells from three independent rat embryos). These results show the presence of functional synaptic sites in our culture model of highly pure enteric neurons.

Next, to study the functional synaptic connectivity of cultured neurons, patch clamp was performed as a whole-cell configuration to measure miniature post-synaptic currents (mPSCs) at D7 (Figures 6F, G). mPSCs correspond to small currents measured in post-synaptic neurons that have been induced by the release of synaptic vesicles from pres-ynaptic terminals. We found that 60% of neurons spontaneously received mPSCs from other neurons. The measured mPSCs showed an average frequency of 2.18 ± 0.41 Hz and an amplitude of 9.25 ± 1.10 pA. The properties of the mPSCs are listed in Table 2. These data indicate that highly pure enteric neurons in culture establish a functional synaptic connectivity.

**TABLE 2:** Summary of miniature post-synaptic currents properties of enteric neurons recorded in whole-cell patch clamp configuration.

Miniature post-synaptic currents			
	**MEAN**	**SEM**	**N**
Frequency (Hz)	2.18	0.41	10
Amplitude (pA)	9.25	1.10	10
Rise (ms)	6.24	0.23	10
Decay (ms)	25.23	1.38	10
10–90 Rise (ms)	4.76	0.23	10
Half Width (ms)	4.36	1.04	10
Rise 50 (ms)	1.54	0.21	10
10–90 Slope	−3.03	0.71	10

### The neuronal enteric network activity

**FIGURE 7:**
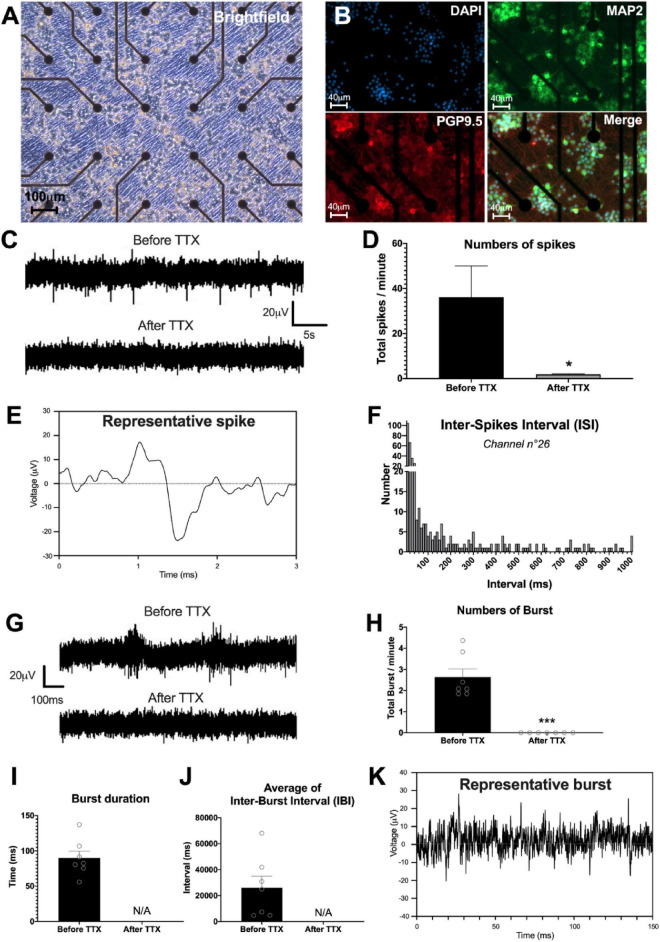
Enteric neuronal network activity. **(A–K)** Analysis of neural network activity of highly pure cultures of enteric neurons by multi-electrode array (MEA) at D28. **(A,B)** Representative image of enteric neurons in MEA, **(A)** brightfield and **(B)** double immunostaining of enteric neurons at D28 with neuronal marker PGP 9.5 (red), MAP2 (green) and the cell nucleus marker DAPI (blue). **(C)** Representative voltage recording at one electrode, showing spontaneous firing and its inhibition by TTX treatment. **(D)** Numbers of spikes per minutes before and after TTX, average of 15 electrodes. **(E)** Detail of a representative enteric neuron spike measured by MEA. **(F)** Histogram representing an example of Inter-Spike Interval (ISI) measured on one electrode (n°26). **(G)** Single electrode recordings showing examples representative traces of two consecutive burst before and after TTX treatment. **(H–J)** Different parameters of burst measured by MEA before and after addition of TTX in the culture, i.e., **(H)** numbers, **(I)** duration, and **(J)** average of inter-burst interval. **(K)** Detail of a representative burst trace measured by MEA. Results were compared using t-test (*p < 0.05; ***p < 0.001). Data are presented as mean ± SEM of *n* = 3 chips from 3 independent rat embryos.

To study the neuronal network activity, we applied the MEA approach to our culture model. The spontaneous activity of the neuronal network was studied at D28 by measuring the field potentials with 60 electrodes on a multi-electrode array chip (Figure 7). First, our results showed that enteric neurons cultured on the MEA chip developed similarly to those in a regular culture plate and were maintained for 28 days of culture. Enteric neurons were grouped into clusters and expressed dendritic (MAP2) and neuronal (PGP9.5) markers (Figures 7A, B).

The study of electrical activity by MEA showed the presence of spontaneous spikes on all 59 electrodes (excluding ref. electrode). For the rest of the analysis, we only considered the most responsive electrodes, defined by at least 6 spikes per minute, which represented an average of 15.3 ± 1.45 electrodes per chip. To investigate whether the spikes originated from neuronal activity, we used TTX to block voltage-dependent Na^+^ channel activity. Before TTX, the number of spikes per minute was 36.2 ± 13.8 on the 15 selected electrodes (Figures 7C, D). The addition of TTX to the culture medium significantly reduced the number of spikes per minute to 1.9 ± 0.2 (*p* < 0.05) (Figures 7C, D). Detailed analysis of the spikes indicated that the spike amplitude is approximately 40 μV, with a duration of approximately 1 ms (Figure 7E). The distribution of the inter-spike interval (ISI), that is, the time that elapses between two spikes, was mostly between 10 and 50 ms (Figure 7F). Finally, we measured burst firing, defined by a pattern of grouped spikes followed by rest periods much longer than the ISI. The data showed that the number of bursts per minute was on average 2.63 ± 0.38 with a duration of 89.97 ± 9.80 ms (Figures 7G–K). In addition, the average of the inter-burst interval was 26.09 ± 8.86 s (Figure 7J). Interestingly, addition of TTX to the medium inhibited the occurrence of bursts (Figures 7G–J). Finally, the burst network analyses did not show any burst synchronization at D28. Spontaneous activity measured by MEA showed the presence of spikes and bursts suggesting the establishment of a functional enteric neuronal network in culture.

## Discussion

In this study, we showed that highly pure primary culture of enteric neurons could be obtained from the guts of rat embryos. The implemented protocol resulted in approximately 99.7% purity for enteric neurons, with minimal contribution from muscle cells and virtually no enteric glial cells. We found morphological and phenotypic diversity characteristics of enteric neurons and demonstrated their functionality using several approaches. Electrical excitability and spontaneous neuronal activity were demonstrated using the patch-clamp, VSD, and Ca^2+^ imaging. Furthermore, these highly pure enteric neuronal cultures were able to establish functional synaptic connectivity, as assessed at the single-cell level by patch clamp, and a functional neuronal network, as assessed by MEA. This novel enteric neuron culture model provides a valuable tool for studies targeting cell-autonomous mechanisms involved in the establishment and maintenance of enteric neuron connectivity.

We present a culture model of enteric neurons whose main feature is composed of more than 99% of neurons as early as 7 days of culture, which can be maintained for long-term studies up to a month. Neuronal enrichment was achieved using a protocol based on the three main experimental elements. First, the addition of GDNF to the day of gut explant and dissociated cell seeding is critical for promoting neuronal cell differentiation and survival. Second, the use of glia-conditioned neurobasal medium also supported neuronal enrichment, in accordance with the reported role of EGC in neuroprotection (Abdo et al., 2010) and neuronal maturation of enteric neurons (Le Berre-Scoul et al., 2017). Third, the use of the antimitotic agent AraC, which is known to inhibit cell proliferation, contributed to the elimination of muscle and enteric glial cells. Most current culture models with enteric neurons are primary cultures of dissociated cells derived from embryonic or adult intestines (Saffrey et al., 1991; Hanani et al., 1994; Heuckeroth et al., 1998; Fex Svenningsen et al., 2003; Chevalier et al., 2008; Gomes et al., 2009; Schonkeren et al., 2022) and organotypic cultures of gut explants (Jessen et al., 1983; Natarajan et al., 1999), in which enteric neurons are in the minority among EGCs, smooth muscle cells, fibroblasts, and epithelial cells. This cellular diversity, which recapitulates, in part, the intestinal cellular microenvironment, makes it extremely difficult to study the intrinsic properties of enteric neurons and their ability to directly respond to various stimuli, including gut cell-derived factors. To circumvent this pitfall, several methodologies have been developed in an attempt to enrich gut-derived cultures with enteric neurons; however, isolation and culture of primary enteric neurons is a difficult process and has yielded only a small number of neurons. Myenteric ganglion explants from newborn guinea pigs subjected to antimitotic agent and antibody complement-mediated cytolysis resulted in the elimination of glial cells and therefore enrichment in enteric neurons, but with low yield and a sparse neuronal network (Bannerman et al., 1988). In addition, based on the use of antimitotic compounds, we previously developed a neuron-enriched primary culture from rat embryonic intestines, grown as an indirect co-culture with enteric glial cells (Le Berre-Scoul et al., 2017). Other procedures have used immunoselection of p75NTR-positive cells to isolate neuronal precursors from embryonic and postnatal intestinal tissues (Wu et al., 1999; Anitha et al., 2008; Sato and Heuckeroth, 2008; Gisser et al., 2013; Hawkins et al., 2013). However, all the aforementioned methodologies are relatively complex, often resulting in a sparse neuronal network, and in some cases, leading to neurons with deficient electrical excitability or to cultures containing muscle cells after several days in culture. The methodology described in this study resulted in a primary culture model highly enriched in enteric neurons with a functional neuronal network and was achieved through a relatively simple procedure. This novel model represents an innovative tool for deciphering the cell-autonomous mechanisms or pathways involved in the formation and functionality of enteric neuron networks. Moreover, it offers the possibility to establish multi-compartment systems combining highly pure enteric neurons with other pure gut cell cultures, such as epithelial, glial or muscle cells, to study functional interplay between enteric neurons and other cell types.

The implementation of this enteric neuron culture model has led to significant technological advances. First, we provided evidence that lipid-mediated transfection can be achieved in cultured enteric neurons. Although the efficiency of the lipofection methodology used in this study is low, as previously reported for central neurons (Karra and Dahm, 2010), it offers the advantages of being technically simple and requiring regular plasmid vectors without the need to generate virus-based constructs. This lipofection-based approach opens up novel strategies to study the impact of any selected protein on enteric neuron activity and connectivity. Another technical contribution of this novel model of cultured enteric neurons is its applicability to MEA. Multi-electrode array allows simultaneous monitoring of the electrophysiological activity of neuronal circuits at many sites through non-invasive (i.e., extracellular) methods and, therefore, to document the functional properties of neuronal networks. This technique requires pure neuronal culture because the presence of other cell types, such as muscle cells, can prevent contact between the MEA chip electrodes and the cultured neurons, and therefore reduce or interfere with the recorded electrical signal. The enteric neuron culture model developed in this study enables the use of MEA, thus providing a novel functional approach of characterizing neuronal network activity.

We provided morphological and functional evidence that enteric neurons obtained by the methodology described in this study possess properties relevant to physiological conditions. First, morphological analyses of GFP-transfected neurons revealed morphological characteristics reminiscent of Dogiel type I and II neurons, the two main classes of enteric neurons widely described in rats, mice, and the human gastrointestinal tract during postnatal development and adulthood (Cornelissen et al., 1999; Nurgali et al., 2004; Brehmer, 2021). In addition, the neurochemical coding analyses indicated that 17 and 20% of the cultured neurons were cholinergic and nitrergic neurons, respectively. These results are similar to those previously described in other ENS culture models (Le Berre-Scoul et al., 2017; Aubert et al., 2019) and in the colons of young or adolescent rats and mice (Aubert et al., 2019; Parathan et al., 2020), but is lower than the proportions of cholinergic and nitrergic neurons described in adult rodents (Soret et al., 2010). A time-dependent increase in the percentage of cholinergic and nitrergic neurons have been described during the postnatal development in rat colons (de Vries et al., 2010). Thus, our enteric neuron culture model, as well as other ENS culture models, likely represent dynamic systems during development rather than adult stage models. We also showed that cultured neurons are responsive to cholinergic and purinergic agonist stimulation, indicating that they express functional neuroreceptors. They were also able to display spontaneous neuronal activity, as measured by Ca^2+^ imaging, characterized by three major patterns of Ca^2+^ transients: isolated, moderately clustered, and tightly clustered transients, as previously observed in mouse cholinergic myenteric ganglion neurons (Margiotta et al., 2021). The functional significance of these three Ca^2+^ transient patterns remain unknown but may underlie different neuronal subclasses and levels of activity. Interestingly, different neuronal activity patterns of cholinergic and nitrergic neurons, defined by spontaneous Ca^2+^ transients, have been correlated with the contraction and inhibition of the colon (Gould et al., 2019). Finally, we found that isolated enteric neurons were excitable, generating electrically induced and spontaneous APs, as shown by the patch-clamp technique and VSD imaging. Remarkably, the frequency of spontaneous AP measured by these two approaches was similar, suggesting that the same events could be detected by these two distinct methods. Furthermore, the electrophysiological AP properties determined by patch clamp display similar characteristics to those obtained in mixed cultures containing enteric neurons, enteric glial cells, and muscle cells (Bodin et al., 2021) and in short-term cultures of dissociated embryonic intestines (Hao et al., 2011, 2012), suggesting that the intrinsic electrical properties of enteric neurons are retained even without the physiological cellular microenvironment.

We demonstrated that cultured enteric neurons could establish synaptic connections and organize functional neuronal networks. The juxtaposition of the presynaptic marker synapsin 1 clusters to the post-synaptic molecule PSD95 clusters supports the existence of synapses composed of pre- and post-synaptic structures with distinct molecular profiles. Furthermore, using FM1-43, we showed the presence of active synapses that could be classified into three populations according to the fraction of FM1-43 destaining. Interestingly, two patterns of FM1-43 destaining were described in hippocampal synapses, as potential indicator of two distinct modes of exocytosis (Richards et al., 2005). Similarly, also enteric neurons show a stimulus-dependent diversity of their synaptic contacts (Vanden Berghe and Klingauf, 2007), which could explain why in our rat culture experiments different levels of synaptic destaining are observed. Functional synaptic connectivity was also demonstrated in mPSCs, characterized by patch clamp analyses, which showed features similar to those previously observed in mixed ENS cultures (Bodin et al., 2021). Neuronal network activity was also functionally addressed by MEA recordings, which showed spontaneous network activity patterns characterized by single spikes and bursting events. Both types of signals were abolished by TTX, indicating that they were driven by neuronal action potentials (Zhou et al., 2009; Roberts et al., 2019). Multi-electrode array recordings have never been performed directly to enteric neurons but have been used on ileal whole-muscle layer containing the myenteric plexus to measure the oscillating electrical activity (Taniguchi et al., 2013). In addition, MEA have been used on CNS-derived cultured neuronal networks, including cortex, hippocampus, and motor neurons, which showed profiles of spontaneous spiking and bursting activity similar to those obtained in our enteric neuron model (Arnold et al., 2005; Wainger et al., 2014; Negri et al., 2020). Analyses of MEA recordings provided important information for studying the formation, organization, and maintenance of neuronal networks. In particular, bursts corresponding to a fast series of spikes generated by connected neurons are important markers of network formation and activity (Zeldenrust et al., 2018). Interestingly, within the GI tract, neuronal imaging associated with electrophysiology during rhythmic neurogenic motor activity in the mouse colon revealed that large populations of enteric neurons fire in coordinated and repetitive bursts, generating muscle contractions that underlie migrating motor complexes (Spencer et al., 2018). In addition, several recent studies have highlighted the importance of ENS network architecture and functional connectivity in the neurogenic control of patterned motor function of the GI tract (Sasselli et al., 2013; Li et al., 2019; Nestor-Kalinoski et al., 2022). Thus, the application of a highly pure enteric neuronal network culture model to MEA might represent a valuable novel tool to better understand the mechanisms underlying the neuronal network activity and information processing of the ENS.

Our results indicate that these highly pure cultures of enteric neurons exhibited spontaneous electrical activity, which might play a role in neuronal network formation. It has been shown in the CNS and ENS that early spontaneous electrical activity plays a crucial role in the initiation of various processes, such as neuronal differentiation and maturation, and axon growth and formation of synaptic connections (Borodinsky et al., 2012; Hao et al., 2013). Spontaneous neuronal activity was observed in mouse embryonic enteric neurons at E11.5, with an increase in activity until E18.5, followed by a decrease in activity in adulthood. Several channels might be involved, such as voltage-gated Na + channels, N-type voltage-gated Ca^2+^ channels, and purinergic P2 receptors (Hao et al., 2011, 2012, 2017). Further studies are required to characterize the mechanisms and impact of spontaneous electrical activity on network formation in our culture model.

In conclusion, this study describes a novel *in vitro* model of enteric neurons in rats. A major innovation of this model is its high enteric neuronal purity, which can form a complex and functional network. Moreover, its applicability to MEA represents a novel approach for studying the cell-autonomous mechanisms involved in the establishment and maintenance of enteric neuron networks.

## Data availability statement

The original contributions presented in this study are included in the article/supplementary material, further inquiries can be directed to the corresponding author.

## Ethics statement

This animal study was reviewed and approved by the French Standard Ethical Guidelines for Laboratory Animals (Approval number E44015).

## Author contributions

MC and HB: conceptualization. MC, JM, SN, GL, VP, MN, and HB: methodology. MC, ML, AD-G-d-L, CL, and VP: investigation. MC and ML: visualization. GL, MN, and HB: supervision. MC, ML, AD-G-d-L, and HB: writing—original draft. MC, GL, VP, MN, and HB: writing—review and editing. All authors contributed to the article and approved the submitted version.
